# The usefulness of presepsin in the diagnosis of postoperative infectious complications after gastrectomy for gastric cancer: a prospective cohort study

**DOI:** 10.1038/s41598-022-24780-8

**Published:** 2022-12-09

**Authors:** Yoshiro Imai, Ryo Tanaka, Kotaro Honda, Kentaro Matsuo, Kohei Taniguchi, Mitsuhiro Asakuma, Sang-Woong Lee

**Affiliations:** 1 Departments of General and Gastroenterological Surgery, Osaka Medical and Pharmaceutical University, 2-7 Daigaku-Machi, Takatsuki, Osaka 569-8686 Japan; 2 Department of Translational Research Program, Faculty of Medicine, Osaka Medical and Pharmaceutical University, 2-7 Daigaku-Machi, Takatsuki, Osaka 569-8686 Japan

**Keywords:** Biomarkers, Gastroenterology

## Abstract

This prospective study aimed to evaluate presepsin use as a biomarker of on postoperative infectious complications after gastrectomy, compared to C-reactive protein (CRP), white blood cells (WBCs), and neutrophils (Neuts). Overall, 108 patients were enrolled between October 2019 and December 2020. Presepsin, CRP, WBC, and Neut levels were measured preoperatively and on postoperative days (PODs) 1, 3, 5, and 7, using a postoperative morbidity survey. Grade II or higher infectious complications occurred in 18 patients (16.6%). Presepsin levels on all evaluated PODs were significantly higher in the infectious complication group than in the non-complication group (*p* = 0.002, *p* < 0.0001, *p* < 0.0001, and *p* = 0.025, respectively). The area under the curve (AUC) values were the highest for presepsin on PODs 3 and 7 (0.89 and 0.77, respectively) and similar to that of CRP, with a high value > 0.8 (0.86) on POD 5. For presepsin, the optimal cut-off values were 298 pg/mL (sensitivity, 83.3%; specificity, 83.3%), 278 pg/mL (sensitivity, 83.3%; specificity, 82.2%), and 300 pg/mL (sensitivity, 83.3%; specificity, 82%) on PODs 3, 5, and 7, respectively. Presepsin levels on PODs 3, 5, and 7 after gastrectomy is a more useful biomarker of postoperative infectious complications compared to CRP, WBCs, and Neuts, with a high sensitivity and specificity.

## Introduction

Gastric cancer is the fourth leading cause of cancer-related deaths and the fifth most common cancer worldwide^1^. The incidence and mortality of gastric cancer are increased in men compared to those in women^2^. Gastrectomy with lymph node dissection is the only method for curing gastric cancer^3^. The standardization of surgical techniques and perioperative management, as well as improved instrumentation, have led to a low mortality of approximately 1.5% according to the National Clinical Database (NCD)^4^ in Japan. However, the morbidity rates are still high, at 14.2% for distal gastrectomy^5^ and 21.5% for total gastrectomy^6^. Postoperative complications of gastric cancer reduce long-term prognosis^7^, and high postoperative C-reactive protein (CRP) levels are related to poor prognosis^8^. Therefore, the early detection of infectious complications at a clinically asymptomatic stage is important to prevent serious complications.

CRP is often used as an inflammatory biomarker because of its simplicity, versatility, and low cost^9^. However, the CRP levels do not increase in the early stages of infection and have the disadvantage of a slow peak or half-life^10^. In addition, the CRP levels are elevated in various inflammatory conditions other than infection, as well as in invasion such as surgery and trauma, thus making it difficult to determine whether they indicate the presence of infection in clinical practice.

Presepsin, which is the soluble fraction of cluster of differentiation 14 (CD14), is thought to be associated with infections^11^, based on the fact that a subtype of CD14 is present inside and on the cell membranes of macrophages, monocytes, and granulocytes, and is responsible for intracellular transduction of endotoxin signals. Presepsin is a novel biomarker for infection and sepsis. The presepsin levels are increased early in the development of sepsis^12,13^ and are less sensitive to invasion, such as trauma^14^. However, few reports have examined the usefulness of presepsin as a biomarker for infectious complications after gastrointestinal surgery^15–17^, and there have been no relevant reports following gastrectomy.

In laparoscopic sleeve gastrectomy for bariatric surgery, presepsin was more useful for gastric leaks than CRP, white blood cells (WBCs), and neutrophils (Neuts), but not for gastric cancer^18^. However, there have been reports on presepsin as a biomarker of postoperative infectious complications for other cancer types or gastric interventions. Therefore, we hypothesized that presepsin would be useful for infectious complications after gastrectomy for gastric cancer.

This study aimed to evaluate the usefulness of presepsin for determining infectious complications after gastrectomy for gastric cancer, compared with CRP, WBCs, and Neuts.

## Results

### Patients’ characteristics and morbidity

In total, 108 patients who underwent gastrectomy were enrolled in this study. Of these, 75 were men and 33 were women, with a median age of 71 years. The American Society of Anesthesiologists (ASA) scores, surgical dates, and pathological characteristics are shown in Table 1.

**Table 1:** Patients’ characteristics.

Characteristic	N = 108
Age, years	71.5 ± 10.6
Sex, male/female	75/33
**ASA score**	
1/2/3	25/71/12
**Surgical procedure**	
DG/PG/TG/RTG	74/14/13/7
**Reconstruction procedure**	
BI/BII/EG/RY/DT	42/5/13/47/1
**Surgical approach**	
Open/laparoscopic/robotic	17/75/16
**Lymph node dissection**	
D1/D1 + /D2/D2 +	11/60/29/8
Intraoperative bleeding (mL)	54.4 ± 112.8
Operative time (min)	278 ± 85.7
Infectious complications (CD ≥ 2), n (%)	18 (16.6%)
**pStage**	
I/II/III/IV	61/19/24/4

ASA, American Society of Anesthesiologists; DG, distal gastrectomy; PG, proximal gastrectomy; TG, total gastrectomy; RTG, remnant total gastrectomy; BI, Billroth I; BII, Billroth II; EG, esophagogastrostomy; RY, Roux-en-Y; DT, double tract; CD, Clavien–Dindo.

Data are presented as n (%) or mean ± standard deviation.

Infectious complications occurred postoperatively in 18 patients (16.6%) (Table 2). Anastomotic leakage was the most commonly observed complication (n = 7), followed by abdominal abscess (n = 4), pancreatic fistula (n = 3), pneumonia (n = 2), cholecystitis (n = 1), and urinary tract infection (n = 1). The median time of infectious disease diagnosis after gastrectomy was 5.8 days.

**Table 2:** Type of infectious complications.

Infectious complications, n	18
Anastomotic leak	7 (38.8%)
Abdominal abscess	4 (22.2%)
Pancreatic fistula	3 (16.6%)
Pneumonia	2 (11.1%)
Cholecystitis	1 (5.5%)
Urinary tract infection	1 (5.5%)

Data are presented as n (%).

When the patients’ characteristics were compared between the complication and non-complication groups, there were no significant differences in the patients’ background characteristics, including age, sex, ASA score, or surgical date (Table 3). Regarding surgical factors, only the amount of blood loss was higher in the complication group than in the non-complication group.

**Table 3:** Comparison of patients’ characteristics between the postoperative infectious complication and non-complication groups.

	Non-Complication (n = 90)	Complication (n = 18)	*p* value
Age, years	70.9 ± 11.2	74.7 ± 6.6	0.22
Sex, male/female	69/21	14/4	0.4
**ASA score**			
1/2/3	23/57/10	2/14/2	0.4
**Surgical procedure**			
DG/PG/TG/RTG	63/11/9/7	11/3/4/0	0.3
**Reconstruction procedure**			
BI/BII/EG/RY/DT	33/4/10/42/1	9/1/3/5/0	0.19
**Surgical approach**			
Open/laparoscopic/robotic	14/63/13	3/12/3	0.95
**Lymph node dissection**			
D1/D1 + /D2/D2 +	10/50/23/7	1/10/6/1	0.82
Intraoperative bleeding (mL)	50.1 ± 113	75 ± 109	0.03
Operative time (min)	276 ± 85.7	284 ± 87.8	0.45
**pStage**			
I/II/III/IV	52/16/18/4	9/3/6/0	0.53

ASA, American Society of Anesthesiologists; DG, distal gastrectomy; PG, proximal gastrectomy; TG, total gastrectomy; RTG, remnant total gastrectomy; BI, Billroth I; BII, Billroth II; EG, esophagogastrostomy; RY, Roux-en-Y; DT, double tract.

### Biomarker levels

**Figure 1:**
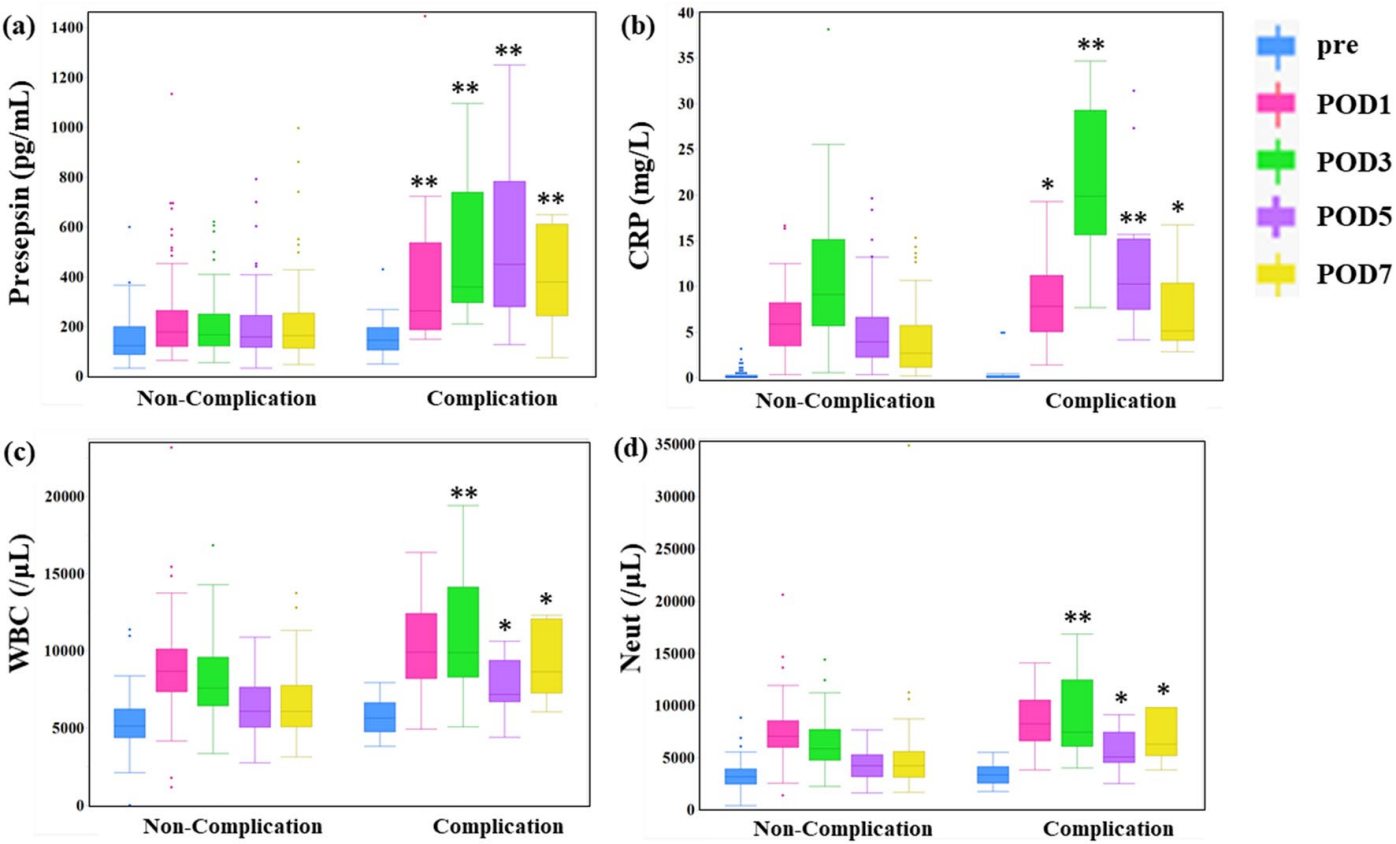
Box plot showing perioperative changes of each biomarker. (**a**) Presepsin, (**b**) CRP, (**c**) WBC, (**d**) Neut. Left side, non-complication group; right side, complication group; CRP, C-reactive protein; WBC, white blood cells; Neut, neutrophils. **: *p* < 0.01; *: *p* < 0.05.

The box plot of each biomarker in the perioperative change is shown in Fig. 1a–d. In the 90 patients with no infectious complications, median presepsin levels were 157 pg/mL (25th to 75th percentile: 33–707 pg/mL) and 180 pg/mL (64–1134 pg/mL), 169 pg/mL (56–621 pg/mL), 159 pg/mL (35–792 pg/mL), and 165 pg/mL (48–997 pg/mL), preoperatively and on PODs 1, 3, 5, and 7, respectively. The postoperative values were the same as the preoperative values. In the 18 patients with postoperative infectious complications, the median presepsin levels were 161 pg/mL (25th to 75th percentile: 50–430 pg/mL) and 263 pg/mL (150–1446 pg/mL), 360 pg/mL (211–1097 pg/mL), 450 pg/mL (128–1251 pg/mL), and 380 (76–650 pg/mL), respectively. Preoperative measurements of the presepsin levels were not significantly different between the non-complication and complication groups (*p* = 0.45). However, presepsin levels on PODs 1, 3, 5, and 7 (*p* = 0.002, *p* < 0.0001, *p* < 0.0001, and *p* = 0.025, respectively) were significantly higher in the complication group compared to the non-complication group (Table 4).

**Table 4:** Comparison of changes in Presepsin, CRP, WBC, and Neut levels, between the infectious complication and non-complication groups.

	Non-complication (n = 90)	Complication (n = 18)	*p* value	Non-complication (n = 90)	Complication (n = 18)	*p* value
	Presepsin level (pg/mL)			CRP level (mg/dL)		
Preoperatively	157 (33–707)	161 (50–430)	0.45	0.08 (0.01–3.16)	0.06 (0.01–4.9)	0.77
POD 1	180 (64–1134)	263 (150–1446)	0.002	5.9 (0.3–16.4)	7.8 (1.4–19.2)	0.03
POD 3	169 (56–621)	360 (211–1097)	< 0.0001	9.1 (0.5–38.1)	19.8 (7.6–34.6)	< 0.0001
POD 5	159 (35–792)	450 (128–1251)	< 0.0001	3.9 (0.3–19.6)	10.2 (4.1–31.4)	< 0.0001
POD 7	165 (48–997)	380 (76–650)	0.025	2.7 (0.2–15.3)	5.1 (2.8–16.7)	0.03
	WBC count (/μL)			Neut count (/μL)		
Preoperatively	5145 (3548–11 380)	5645 (3810–7940)	0.24	3219 (424–8842)	3366 (1795–5558)	0.45
POD 1	8690 (1170–23 160)	9930 (4930–16 380)	0.06	7068 (1413–20 612)	8246 (3845–14 087)	0.09
POD 3	7580 (3350–16 830)	9905 (5090–19 400)	0.003	5895 (2269–14 406)	7471 (4026–16 878)	0.0002
POD 5	6105 (2750–10 880)	7170 (4420–10 620)	0.01	4277 (1677–7710)	5078 (2537–9123)	0.012
POD 7	6105 (2750–10 880)	7170 (4420–10 620)	0.01	4251 (1692–34,897)	6307 (3848–9856)	0.01

POD, postoperative day; CRP, C-reactive protein; WBC, white blood cell; Neut, neutrophil.

CRP, WBC, and Neut levels, unlike presepsin, increased and then decreased postoperatively, regardless of infectious complications. CRP and presepsin levels were not significantly different preoperatively between the two groups (*p* = 0.77), but were significantly higher in the complication group than in the non-complication group on PODs 1, 3, 5, and 7 (*p* = 0.03, *p* < 0.0001, *p* < 0.0001, *p* = 0.03, respectively). WBC and Neut levels showed no significant differences between the complication and non-complication groups preoperatively (*p* = 0.24 and 0.45, respectively) and on POD 1 (*p* = 0.06 and 0.09, respectively), but were significantly higher in the complication group than in the non-complication group on PODs 3 (*p* = 0.003 and 0.0002, respectively), 5 (*p* = 0.01 and 0.012, respectively), and 7 (*p* = 0.01 and 0.01, respectively).

### Receiver operator characteristics analysis of the biomarker

**Figure 2:**
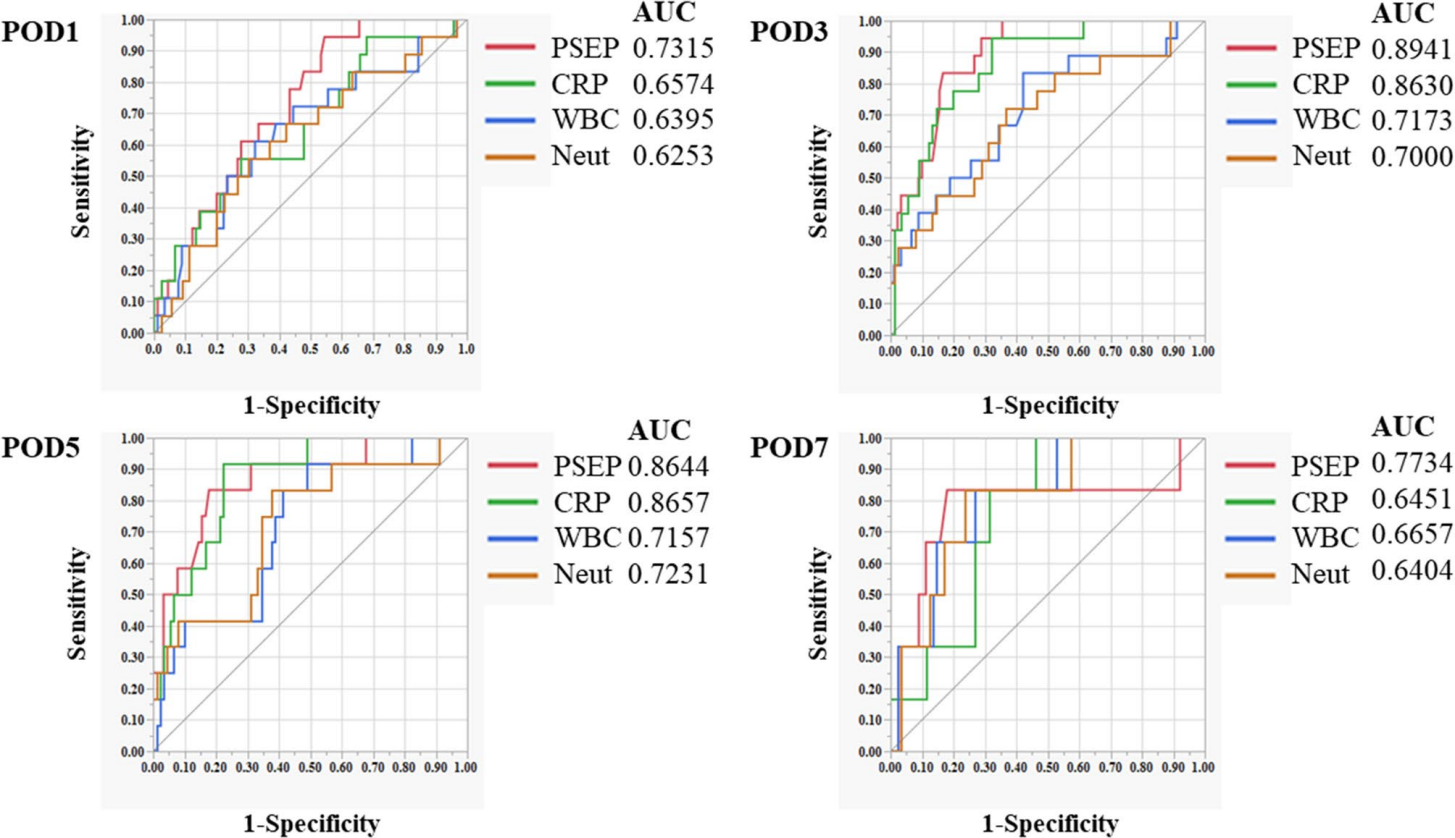
AUCs of presepsin, CRP, WBC, and Neut according to the ROC curve. (**a**) POD 1, (**b**) POD 3, (**c**) POD 5, (**d**) POD 7. ROC, receiver operating characteristic; CRP, C-reactive protein; WBC, white blood cells; Neut, neutrophils; POD, postoperative day; AUC, area under the curve.

We compared the AUC values of presepsin, CRP, WBC, and Neuts, on PODs 1, 3, 5, and 7 between the groups (Fig. 2a–d). The AUC values in the pooled prediction model were comparable to those considered separately. The AUC values were highest for presepsin on PODs 1, 3, and 7 (0.731, 0.89, and 0.77, respectively). On POD 5, the AUC value of presepsin was similar to that of CRP, with a high value > 0.8 (0.86). The AUC values of presepsin were > 0.7 on all days, including on PODs 1, 3, 5, and 7.

Then, the ROC curve was used to calculate the cut-off value for each infectious complication of the PODs (Table 5). On POD 1, the sensitivity and positive predictive value (PPV) of presepsin exceeded 90%, but the specificity and negative predictive value (NPV) were generally as low as 50%. For CRP, WBCs, and Neuts, all values for sensitivity, specificity, the PPV, and NPV were low at 50–70%. On PODs 3, 5, and 7, all the values for sensitivity, specificity, the PPV, and NPV exceeded 80% for presepsin. However, CRP and WBCs showed sensitivity and a PPV that exceeded 80% on PODs 3, 5, and 7, and Neuts showed sensitivity and a PPV exceeding 80% on PODs 5 and 7. For the other parameters, except for presepsin, specificity and NPV did not exceed 80% at any time postoperatively.

**Table 5:** Diagnostic accuracy of presepsin, CRP, WBCs, and Neuts for postoperative infectious complications on PODs 3, 5, and 7.

POD 1	Cut-off value	Sensitivity	Specificity	PPV	NPV
Presepsin	170	94.4	54.6	94.4	45.5
CRP	7.68	55.5	72.3	55.5	72.2
WBCs	9630	61.1	67.8	61.1	67.7
Neuts	8093	55.5	70.0	55.5	70.0
**POD 3**					
Presepsin	298	83.3	83.3	83.3	83.3
CRP	12.4	94.4	67.8	94	67.7
WBCs	8280	83.3	57.8	83.3	57.7
Neuts	6577	66.6	63.3	66.6	63.3
**POD 5**					
Presepsin	278	83.3	82.2	83.3	82.2
CRP	7.34	91.6	77.8	91.6	77.7
WBCs	6190	91.6	51.1	91.6	51.1
Neuts	4537	83.3	62.2	83.3	62.2
**POD 7**					
Presepsin	300	83.3	82	83.3	81.1
CRP	2.8	83.3	54	83.3	44.4
WBCs	7680	83.3	73.1	83.3	72
Neuts	5653	83.3	76.4	83.3	75.5

POD, postoperative day; CRP, C-reactive protein; WBCs, white blood cells; Neuts, neutrophils; NPV, negative predictive value; PPV, positive predictive value.

## Discussion

Several meta-analyses have reported that presepsin is a valuable biomarker for sepsis^19–21^. However, there are few reports on the relationship between postoperative infectious complications and presepsin levels^15–17,22,23^. Moreover, there are only two reports on postoperative gastrointestinal surgery^15–17^, and to the best of our knowledge there have been no reports on postoperative gastrectomy. The results of the present study showed, for the first time, that presepsin was useful as a biomarker for determining infectious complications after gastrectomy for gastric cancer, both in terms of sensitivity and specificity. High postoperative CRP levels are related to poor prognosis of gastric cancer ^8^. Therefore, in the early detection of infectious complications, presepsin is useful in improving prognosis because it allows for appropriate therapeutic intervention, thereby contributing in minimizing elevated CRP levels.

Cikot et al. reported that presepsin is a useful biomarker for gastrointestinal anastomotic leakage^16^. However, Cikot et al. revealed that the only intended complication was anastomotic leakage, and the details of the surgical procedure, including the operative time, blood loss, and surgical approach, were unknown. Therefore, the effect of surgical invasiveness remains unknown. In a series of 30 cases, Takeuchi et al. reported that presepsin was an accurate diagnostic marker of postoperative infectious complications after esophagectomy^15^, and they also compared presepsin to procalcitonin preoperatively, immediately post-surgery, and on PODs 1, 2, 3, 5, and 7. Nevertheless, the sample size of the study was small.

In our study, presepsin had some advantages over other parameters, with a sensitivity and specificity > 80% on PODs 3, 5, and 7. However, on POD 1, presepsin showed low specificity (54.6%) and high sensitivity (94.4%). The median number of days of diagnosis of infectious complications was 5.8 days after gastrectomy. Three patients had onset of an infectious complication on POD 3, and none had onset of an infectious complication before that. In addition, seven patients developed infectious complications after POD 7. This supports the low specificity of presepsin on POD 1 because postoperative adverse events had not yet occurred.

Shi et al. reported that CRP levels on PODs 3 and 5 were significantly useful in the diagnosis of infectious complications after gastrectomy^24^. Our results similarly showed that serum levels of CRP on PODs 3 and 5 were useful inflammatory biomarkers, with an AUC value of 0.8 or higher. However, the specificity of presepsin was higher than that of CRP. In the non-complication group, CRP, WBC, and Neut levels were transiently elevated due to the effects of surgical invasion, but the levels of presepsin did not change. Although esophagectomy is one of the most invasive surgical procedures in gastrointestinal surgery, Takeuchi et al. reported that presepsin levels did not change significantly after esophagectomy in the non-complication group^15^. This finding indicates that presepsin expression was not affected by postoperative invasion. Amanai et al. reported that prespsin levels did not change significantly after colorectal surgery but increased with infectious complications, making it a useful marker when used in combination with CRP^17^.

The reference range for presepsin from healthy volunteers assayed using the PATHFAST presepsin assay was 314 pg/mL or less^25^. This value was close to the cut-off value for postoperative infectious complications in our study. Furthermore, the cut-off values remained almost the same for all periods (PODs 3, 5, and 7) and can be interpreted as a risk of postoperative infectious complications if they exceed the reference values. However, the cut-off value of CRP was different for each period, and the cut-off value was much higher than the reference value (< 0.8 mg/mL), making it difficult to determine postoperative infectious complications.

The present study has several limitations that need to be acknowledged. First, this was a single-center study limited to the Japanese population. However, we believe that the infectious complication rate in this study is comparable to that of the NCD in Japan and is consistent with that in the perioperative period of gastrectomy for gastric cancer, as gastrectomy has been performed in recent years with a high percentage of laparoscopic procedures. Second, only presepsin and routine blood test variables, such as CRP, WBC, and Neut levels, were measured, whereas other inflammatory biomarkers, such as procalcitonin, were not. There are reports that procalcitonin is useful for the accurate diagnosis of infectious complications after gastrectomy for gastric cancer^26,27^, but there are no reports directly comparing procalcitonin and presepsin. Presepsin has been reported to be superior to procalcitonin in the diagnosis of infectious complications after esophagectomy for esophageal cancer^15^. Therefore, it is necessary to compare presepsin with procalcitonin in the future, and to examine whether the combined use of these inflammatory biomarkers will improve diagnostic accuracy. Third, the number of patients with infectious complications was low at 18 (16.6%). Since the rate of complications is not high, the cost-effectiveness of routine measurement of presepsin should be further considered. Finally, because of the difference in presepsin levels between the non-complication and complication groups on POD 1, it is crucial to consider whether there are factors other than infectious complications that affect presepsin. Unlike other biomarkers, presepsin is less affected by postoperative invasion^15,17^. No significant differences were found in patients’ background characteristics, including age, sex, ASA score, disease state, or surgical date between the non-complication and complication groups. Therefore, case accumulation and multivariate analysis are required to examine influencing factors.

Presepsin levels on PODs 3, 5, and 7 after gastrectomy for gastric cancer are valuable biomarkers for detecting postoperative infectious complications, compared with other inflammatory biomarkers such as CRP, WBCs, and Neuts. Presepsin is a very simple biomarker that can be used to suspect postoperative infectious complications when the reference value is exceeded, even after gastrectomy, regardless of the number of postoperative days.

## Methods

### Ethics statements

This study protocol was conducted in accordance with the Declaration of Helsinki and its latest amendments. The study protocol was approved by the ethics committee of Osaka Medical and Pharmaceutical University Hospital (approval number: 2020-005). Informed consent was obtained from all patients.

### Patients

This was a single-institution prospective observational study. Between October 2019 and December 2020, 113 patients with gastric cancer underwent curative gastrectomy at Osaka Medical and Pharmaceutical University Hospital in Japan. Two patients who did not give consent for this study, two patients with active infection before gastrectomy, and one patient undergoing emergency surgery for bleeding, were excluded. Therefore, data from 108 patients were analyzed in the present study.

### Measurements and study endpoint

We measured presepsin in addition to CRP, WBCs, and Neuts, which are usually measured in the perioperative period. We examined presepsin by collecting at least 2 mL of blood samples preoperatively and at 1, 3, 5, and 7 days post-gastrectomy, at 7:00 a.m. Presepsin was measured with a fully automated immunoassay analyzer (PATHFAST, LSI Medience Corporation, Tokyo, Japan), according to the manufacturer’s instructions^25^. For patients with infectious complications, the blood samples collected after the onset of complications were not included in the analysis. The primary endpoint was the ability of presepsin to detect postoperative infectious complications in patients who underwent curative gastrectomy for cancer.

### Surgical procedures

Lymph node dissection, surgery, and gastric reconstruction were performed according to the Japanese Gastric Cancer Treatment Guidelines^28^. At our institute, almost all resections for gastric cancer are performed laparoscopically or robotically regardless of the clinical stage, except for clinical trials limited to open surgical procedures and emergency surgery, such as surgery for perforation or acute bleeding. The pathological stage and residual tumor status were confirmed according to the Japanese classification of gastric carcinoma^29^.

### Perioperative management

At our institute, the enhanced recovery after surgery protocol has been introduced in perioperative care^30^. The patients took 250 mL of an oral carbohydrate solution at night before surgery and 2 h before anesthesia. On POD 1, the patients started walking and were allowed to drink clear fluid. The patients started to ingest a liquid diet on POD 2, after which the diet was continued through four daily steps to eventually consume regular food on POD 6. Acetaminophen was administered orally twice daily until POD 5. Prophylactic antimicrobials were administered before incision and 3 h after the first dose during the surgical operation.

### Postoperative complications

Postoperative complications were diagnosed up to 30 days after the gastrectomy. Pancreatic fistula was diagnosed based on the definitions of the International Study Group on Pancreatic Fistula^31^. Anastomotic leakage was diagnosed via a radiological examination using an orally administered contrast medium.

### Statistical analysis

All statistical analyses were performed using JMP Pro 15 (version 15, SAS Institute, Cary, NC, USA). Continuous variables are presented as mean ± standard deviation and were compared using the Wilcoxon rank-sum test. The chi-square test and Fisher exact probability test were used to compare the differences in categorical variables between the complication and non-complication groups. Receiver operating characteristic (ROC) analysis was performed to assess the diagnostic accuracy of infectious complications by evaluating the area under the curve (AUC). An AUC ≥ 0.8 was considered to show high diagnostic accuracy, with those closest to 1 considered to be the most predictive. Statistical significance was set at *p* < 0.05.

## Data availability

The datasets used and/or analysed during the current study available from the corresponding author on reasonable request.

## References

[1] Sung H (2021). Global Cancer Statistics 2020: GLOBOCAN sstimates of incidence and mortality worldwide for 36 cancers in 185 countries. CA Cancer J. Clin..

[2] Yao Q, Qi X, Xie SH (2020). Sex difference in the incidence of cardia and non-cardia gastric cancer in the United States, 1992–2014. BMC Gastroenterol..

[3] Colvin H (2017). Gastroenterological surgery in Japan: the past, the present and the future. Ann. Gastroenterol. Surg..

[4] Hasegawa H (2019). Surgical outcomes of gastroenterological surgery in Japan: Report of the National Clinical Database 2011–2017. Ann. Gastroenterol. Surg..

[5] Kunisaki C (2017). Modeling preoperative risk factors for potentially lethal morbidities using a nationwide Japanese web-based database of patients undergoing distal gastrectomy for gastric cancer. Gastric Cancer.

[6] Kikuchi H (2017). Development and external validation of preoperative risk models for operative morbidities after total gastrectomy using a Japanese web-based nationwide registry. Gastric Cancer.

[7] Tokunaga M, Tanizawa Y, Bando E, Kawamura T, Terashima M (2013). Poor survival rate in patients with postoperative intra-abdominal infectious complications following curative gastrectomy for gastric cancer. Ann. Surg. Oncol..

[8] Kurokawa Y (2020). Prognostic value of postoperative C-reactive protein elevation versus complication occurrence: A multicenter validation study. Gastric Cancer.

[9] Oliveira CF (2013). Procalcitonin versus C-reactive protein for guiding antibiotic therapy in sepsis: a randomized trial. Crit. Care Med..

[10] Vigushin DM, Pepys MB, Hawkins PN (1993). Metabolic and scintigraphic studies of radioiodinated human C-reactive protein in health and disease. J. Clin. Invest..

[11] Shozushima T (2011). Usefulness of presepsin (sCD14-ST) measurements as a marker for the diagnosis and severity of sepsis that satisfied diagnostic criteria of systemic inflammatory response syndrome. J. Infect. Chemother..

[12] Liu B, Chen YX, Yin Q, Zhao YZ, Li CS (2013). Diagnostic value and prognostic evaluation of presepsin for sepsis in an emergency department. Crit. Care.

[13] Ulla M (2013). Diagnostic and prognostic value of presepsin in the management of sepsis in the emergency department: A multicenter prospective study. Crit. Care.

[14] Hoshino K (2017). Incidence of elevated procalcitonin and presepsin levels after severe trauma: a pilot cohort study. Anaesth. Intensive Care.

[15] Takeuchi M (2020). The perioperative presepsin as an accurate diagnostic marker of postoperative infectious complications after esophagectomy: A prospective cohort study. Esophagus.

[16] Cikot M (2018). The importance of presepsin value in detection of gastrointestinal anastomotic leak: A pilot study. J. Surg. Res..

[17] Amanai E (2022). Usefulness of presepsin for the early detection of infectious complications after elective colorectal surgery, compared with C-reactive protein and procalcitonin. Sci. Rep..

[18] Binboga S (2019). Plasma presepsin in determining gastric leaks following bariatric surgery. Turk. J. Biochem..

[19] Zhang X, Liu D, Liu YN, Wang R, Xie LX (2015). The accuracy of presepsin (sCD14-ST) for the diagnosis of sepsis in adults: A meta-analysis. Crit. Care.

[20] Wu J, Hu L, Zhang G, Wu F, He T (2015). Accuracy of presepsin in sepsis diagnosis: A systematic review and meta-analysis. PLoS ONE.

[21] Tong XM, Cao YT, Yu M, Han CW (2015). Presepsin as a diagnostic marker for sepsis: Evidence from a bivariate meta-analysis. Ther. Clin. Risk Manag..

[22] Clementi A (2019). Presepsin and procalcitonin levels as markers of adverse postoperative complications and mortality in cardiac surgery patients. Blood Purif..

[23] Koakutsu T, Sato T, Aizawa T, Itoi E, Kushimoto S (2018). Postoperative changes in presepsin level and values predictive of surgical site infection after spinal surgery: A single-center, prospective observational study. Spine (Phila Pa 1976).

[24] Shi J (2020). Clinical predictive efficacy of C-reactive protein for diagnosing infectious complications after gastric surgery. Therap. Adv. Gastroenterol..

[25] Okamura Y, Yokoi H (2011). Development of a point-of-care assay system for measurement of presepsin (sCD14-ST). Clin. Chim. Acta.

[26] Yang WC (2021). Procalcitonin as an early predictor of intra-abdominal infections following gastric cancer resection. J. Surg. Res..

[27] Thereaux J (2020). A commentary on: diagnostic accuracy of procalcitonin as an early predictor of infection after radical gastrectomy for gastric cancer: A prospective bicenter cohort study. Int. J. Surg..

[28] Japanese Gastric Cancer Association. Japanese Gastric Cancer Treatment Guidelines 2018 (5th edition). *Gastric Cancer* **24**, 1–21 (2021). 10.1007/s10120-020-01042-y PMC7790804 32060757

[29] Japanese Gastric Cancer Association. Japanese classification of gastric carcinoma: 3rd English edition. *Gastric Cancer* English edition **14**, 101–112 (2011). 10.1007/s101209800016 11957040

[30] Tanaka R (2017). Protocol for enhanced recovery after surgery improves short-term outcomes for patients with gastric cancer: a randomized clinical trial. Gastric Cancer.

[31] Bassi C (2005). Postoperative pancreatic fistula: an international study group (ISGPF) definition. Surgery.

